# Can Industrial Collaborative Agglomeration Reduce Haze Pollution? City-Level Empirical Evidence from China

**DOI:** 10.3390/ijerph18041566

**Published:** 2021-02-07

**Authors:** Yunling Ye, Sheng Ye, Haichao Yu

**Affiliations:** 1 School of Economics and Management, Wuhan University, Wuhan 430072, China; 2 Anhui Institute of Industry and Information Technology, Hefei 230001, China; yes@ahjxw.gov.cn; 3 Institute of Central China Development, Wuhan University, Wuhan 430072, China; 4 Institute of Regional and Urban-Rural Development, Wuhan University, Wuhan 430072, China

**Keywords:** haze pollution, industrial collaborative agglomeration, spatial dynamic panel model

## Abstract

We analyze the mechanism for industrial co-agglomeration in Chinese 283 cities to affect haze pollution from 2003 to 2016 and examine the possible mediating effects of urbanization and energy structure between haze pollution and industrial co-agglomeration, finally obtaining the following results. First, industrial co-agglomeration and haze pollution across China, including central and eastern regions keep a typical inverted U-shaped curve relationship. That is, industrial co-agglomeration first promotes haze pollution and then restrains it. However, the impact of industrial co-agglomeration on haze pollution in western China is still on the left side of the inverted U-shaped curve, reflecting a promotion effect. Second, industrial co-agglomeration has a significant spatial spillover effect on haze pollution. Additionally, industrial co-agglomeration can promote haze pollution in local regions but inhibit it in surrounding regions in both the short and long run. In contrast, when the industrial co-agglomeration index exceeds the inflection point (3.6531), it benefits the reduction of haze pollution in local regions, while not being conducive to it in the neighboring regions. Third, industrial co-agglomeration can affect haze pollution through urbanization and energy structure, that is, urbanization and energy structure play an intermediary role between them.

## 1. Introduction

Although mankind has made great progress in industrialization and urbanization globally, the increasing crisis on resource and environmental has been a matter of concern for the society at large [1]. Among them, haze pollution, as one of the most serious environmental pollution problems [2], not only affects the working enthusiasm and production efficiency of laborers, but also significantly reduces atmospheric visibility, causing great inconvenience to the daily lives of residents [3]. Currently, new industrialization is undergoing a stage of rapid expansion in China. However, due to the extensive economic development mode, low energy efficiency, inefficient environmental governance, and other problems, hazy weather occurs frequently [4], which brings a serious health hazard to inhabitant [5]. In the face of the increasing haze pollution, Chinese government has raised the status of ecological environment governance to an unprecedented level. Meanwhile, the public awareness of environmental protection has gradually awakened and the concept of “green development” [6] has become a consensus of society.

In the process of China’s rapid economic development, resource endowments and factor allocations in different regions have not been balanced [7]. In order to pursue knowledge spillover, technology sharing, and maximize their own interests, enterprises with low economic benefits actively approach those with high economic benefits, forming industrial agglomerations in specific regions [8]. Furthermore, with the continuous extension of manufacturing industry chain, producer services run through all links of manufacturing production [9]. A large part of profits created by manufacturing enterprises comes from productive service industry [10]. As an agglomeration economic mode containing both diversification and specialization [11], the co-agglomeration of producer services and manufacturing has been considered to be a norm of industrial economic development in most regions of China. Such as the Beijing-Tianjin-Hebei urban agglomeration, the Pearl River Delta, and the Yangtze River Delta, there have been “professional towns,” “industrial parks,” and “technological parks” with the characteristics of industrial co-agglomeration of producer services and manufacturing [12].

However, although industrial agglomeration exerts positive effects, such as knowledge spillovers and economies of scale, it also causes increasingly serious environmental problems [13]. The Air Quality Index released from the Ministry of Ecology and Environment shows that the main source of China’s haze pollution is PM_2.5_ [14]. PM_2.5_ has small particle size, large surface area, and easy to carry toxic substances in the atmosphere for a long time, so it causes a harmful impact on public health and atmospheric environment [15]. Its emissions are mainly concentrated in the East-Central region north of the Yangtze River, which is also a major manufacturing agglomeration area. Is the simultaneity of a higher level of industrial agglomeration and a higher degree of haze pollution an inevitable result in regional economic development process? Theoretically, the spillover of technological innovation, economies of scale, and centralized treatment of pollution generated by industrial agglomeration reduces the cost of environmental governance. Moreover, the circular economy formed in the agglomeration area also improves the utilization efficiency of energy, thus achieving the improvement of environmental quality [16]. Industrial co-agglomeration belongs to the range of industrial agglomerations. Co-agglomeration of producer services and manufacturing can alleviate industrial isomorphism and excessive competition caused by manufacturing agglomeration [17]. It is also beneficial to improve the level of technical innovation and specialization, realize the sharing and optimal allocation of resources, and accelerate industrial transformation and upgradation [18]. What is the impact on haze pollution from co-agglomeration of producer services and manufacturing? Does this co-agglomeration reduce haze pollution as expected? What is the possible conduction mechanism? Is there a spatial spillover effect and regional difference in the relation between haze pollution and industrial co-agglomeration? The answers to these questions can help optimize China’s industrial structure, provide scientific reference for haze pollution governance.

Based on these factors, this study collects urban panel data in 2003–2016 from 283 Chinese cities to estimate the degree of co-agglomeration of producer services and manufacturing and uses the PM_2.5_ concentration data monitored by satellites to represent haze pollution. Dynamic spatial panel model and mediating effect model are used to analyze the impact of co-agglomeration of producer services and manufacturing on haze pollution and its possible transmission mechanism. The marginal contribution of this study is mainly reflected in the following three aspects. First, most of the existing economic research on China’s air pollution focus on conventional pollutants such as SO_2_ and CO_2_, while the discussion on PM_2.5_, the culprit of haze pollution, is relatively scarce. Furthermore, most studies on haze pollution stay at the provincial level [19], or partial cities [20], it is difficult to comprehensively and effectively describe the haze pollution impact with urban characteristics. In order to fill this gap, we accumulate PM_2.5_ concentration data of 283 Chinese cities from 2003 to 2016 and comprehensively investigate haze pollution from an urban scale with the focus on its spillover effect and spatial correlation. Second, most of previous studies only examined the impact of single industrial agglomeration on haze pollution [21]. Nowadays, the industrial agglomeration of Chinese cities is mainly manifested in the co-agglomeration of producer services and manufacturing. Accordingly, we calculates the industrial co-agglomeration of producer services and manufacturing index of Chinese cities, reveal the relationship in the impact of industrial co-agglomeration on haze pollution and further compare the direct and spillover effects of that in both short and long run. Third, haze pollution is not confined to a specific region [22]. To a large extent, it spreads to surrounding areas due to some economic mechanisms like pollution leakage and industrial transfer, as well as numerous natural factors like atmospheric circulation, which causes an obvious spatial correlation. Therefore, spatial factors need to be considered when using econometric models to study haze pollution [23]. Although the spatial effect can be analyzed by using the spatial econometric model—considering the influence of “path dependence” and ignoring the influence of space-time lag effects can also lead to errors. Consequently, the dynamic spatial panel model [24,25] is used to control the spatial and temporal lag effect of haze pollution, which will enhance the robustness of the analysis results.

The above-mentioned approaches help to summarize the spatial characteristics of haze pollution in China, explore the economic causes of frequent haze weather, and identify the key factors to control haze pollution. This will provide necessary experience support for the rational formulation and effective implementation of policies for controlling haze in China. Equally important, controlling haze pollution contributes to the physical and mental health of public, provides a better living environment for residents, and increases their sense of happiness.

## 2. Literature Review and Theoretical Hypotheses

Up to now, there is no consensus regarding the relationship between environmental pollution and industrial agglomeration. Both the positive and negative externalities of industrial agglomeration determine its environmental effects simultaneously [26,27]. Some studies state that regional environmental pollution is alleviated due to industrial agglomeration. For example, Yan et al. [28] studied environmental pollution and its relationship with industrial agglomeration, drawing the conclusion that, the development of industrial agglomeration contributed to the reduction of environmental pollution in the short term. According to the empirical analysis of Li et al. [29], an improvement of industrial agglomeration level can reduce the emission of industrial pollution in China and alleviate the conflict between industrial development and ecological environment. Moreover, Yoon et al. [30] analyzed the case evidence from the Korean textile industry cluster, showing that producer agglomerations could spawn closed loop production, and the “collective ecological efficiency” can be achieved. Chen et al. [31] used a big panel dataset of Chinese cities, and found that when the level of agglomeration exceeded an inflection point, the agglomeration could generate pollution reduction.

Based on the above analysis, industrial co-agglomeration can have a positive impact on ecological environment through the following four channels. First, with the deepening of industrial co-agglomeration, producer services gradually show the characteristics of low energy consumption and low pollution by leveraging their high-tech and high-added-value attributes. Through the spillover effect of knowledge, the production efficiency and management level of manufacturing industry can be improved significantly [32], which will enhance the industrial correlation effect of manufacturing and producer services, promoting the regional industrial structure more greening [33]. Second, industrial co-agglomeration can form a large-scale specialized labor market in spatial geography, reducing the cost of searching labor for enterprises. In addition, different types of enterprises will gradually form stable industrial structures and market relations, and pay more attention to differentiated competition [34]. This will help to improve resource utilization, alleviate the phenomenon of industrial convergence, and contribute to the governance of environmental pollution. Third, the gathering of a large number of enterprises is conducive to sharing facilities for saving energy and reducing pollution emissions, minimizing the risk of applying “green innovation” technology [35]. Fourth, the scale increase in industrial co-agglomeration is conducive to making pollution disposal more centralized; it not only reduces the operating costs of enterprises, but also promotes the formation of specialized pollution disposal markets. This will help to realize the scale economies effect of environmental governance [36].

However, some researchers believe that industrial agglomeration undermines the improvement of regional environmental quality [37]. For instance, Virkanen [38] considered that water and air pollution in the whole country of Finland was mainly caused by industrial agglomeration in southern regions. Ren et al. [39] analyzed the water quality along the Huangpu River in Shanghai, believing that the rapid deterioration of water quality in the Huangpu River was mainly due to the expansion of production scale caused by industrial agglomeration. Zhang et al. [40] believed that industrial agglomeration acted on environmental pollution through output scale, industrial structure and production efficiency, and that the output scale was the main cause of environmental pollution. Additionally, Zhang found that eastern coastal cities in China with higher agglomeration levels have the highest haze pollution. Feng et al. [41] pointed out that industrial agglomeration aggravated pollution agglomeration.

According to the literature review, there are three possible channels: First, in the initial stage of co-agglomeration of producer services and manufacturing industries, manufacturing accounts for a relatively high proportion. The continuous aggregation of producer services effectively reduces the production cost and facilitates the flow of human capital, information, and technology in the agglomeration area [42]. Most of the growing enterprises, because of their weak awareness of environmental protection, choose extensive development to obtain economic benefits in the process of mutual competition. Therefore, the improvement of industrial innovation ability is mainly used to expand production capacity rather than reduce pollution emissions. This will aggravate environmental pollution [43]. Second, the scale of agglomeration is relatively small in the early stage. The optimal level of resource allocation has not been realized in the market, which increases the cost of environmental pollution control and aggravates haze pollution [44]. Third, a simple clustering of industries is likely to be dominated by low-end manufacturing and traditional productive service industries with high pollution emission and energy consumption. The lower level of industrial co-agglomeration with lower level is not conducive to effective cooperation between producer services and manufacturing industries. The formation of this “inefficient equilibrium” [45] is an inefficient duplication of production activities in various industries.

**Figure 1:**
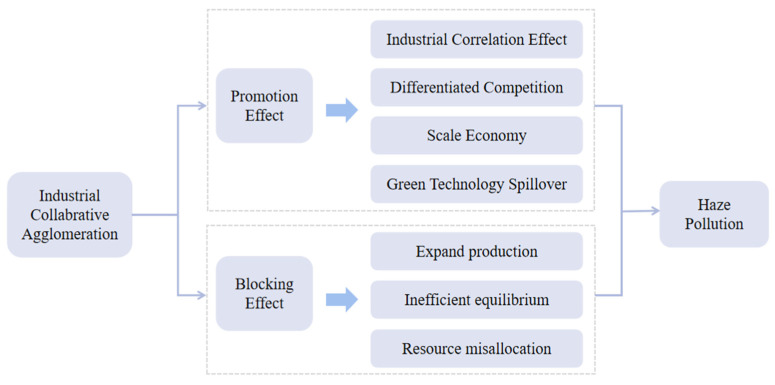
Mechanism for industrial co-agglomeration to affect haze pollution.

Figure 1 shows the impact mechanism of industrial co-agglomeration on haze pollution. Combined with the above analysis, this study infers that industrial co-agglomeration brings an impact to haze pollution, probably in a nonlinear form. Therefore, the theoretical hypothesis below is put forward in this study:

**Hypothesis** **1.** *There may be an inverted U-shaped curve correlation between industrial co-agglomeration and haze pollution. That is, industrial co-agglomeration firstly promotes the haze pollution, but after exceeding an inflection point, it inhibits the haze pollution*.

In the process of co-agglomeration between the producer services and manufacturing industries, regions with higher population concentration and better economic development can transfer talents, technology, and knowledge to surrounding areas through spillover effect. Hence, the economically space-time backward areas can alleviate haze pollution and improve resource utilization through technological innovation [46]. Meanwhile, since GDP is an important performance evaluation standard in the incentive mechanism, competition among local governments is fierce.

Driven by local economic interests, various regions often adopt strategic competitive behavior [47]. Local governments compete for resources, such as enterprises, capital, and technologies, by leveraging lax environmental regulation standards. Furthermore, undeveloped areas without competitive advantage can only develop high-pollution industries dominated by manufacturing to promote regional economic growth rapidly. In addition to aggravating haze pollution in local regions, this also produces a negative impact on neighboring regions. Liu also held the view that the haze pollution was not only affected by the industrial agglomeration in local, but also surrounding areas [48]. Based on this, we propose the second hypothesis:

**Hypothesis** **2.** *Industrial co-agglomeration affects haze pollution not only in local regions but also their surrounding areas. We expect that industrial co-agglomeration has a promotion impact on haze pollution in local regions, which will weaken haze pollution in the surrounding regions.*

First, the co-agglomeration of manufacturing and producer services has been accompanied by the concentration of capital, labor, and other factors, accelerating the process of urbanization. A higher level of urbanization is conducive to sharing public resources, such as health care, transportation, and education. It can streamline the allocation of resources rationally and improve economic performance and environmental governance efficiency, and reduce air pollution levels [49]. Furthermore, in the process of urbanization, large-scale industrial production, infrastructure construction, and the use of locomotive also increases the demand for cement, steel, and other high-pollution products, thus aggravating environmental pollution [50]. Therefore, industrial co- agglomeration may affect haze pollution through urbanization.

Moreover, industrial co-agglomeration may also affect haze pollution through energy structure. Considering that China’s energy structure is still dominated by fossil fuels, the environmental impact of industrial co-agglomeration also depends on whether it increases fossil fuel consumption. In the initial stage, manufacturing and producer services are usually concentrated in a single space and producer services cannot provide technical and capital support for manufacturing. The expansion of manufacturing production capacity for higher economic efficiency, resulting in increased energy consumption, expanding haze pollution [51]. With the deepening of the industrial co-agglomeration, producer services, which are matched with manufacturing industries, pour into the agglomeration area. Scientific research institutions and financial departments provide effective support for developing manufacturing industries. The research activities of environment-friendly technologies, processes, and equipment in the manufacturing industry have been carried out. The use of new energies and clean technologies can effectively improve the energy efficiency of the manufacturing industry, change the coal-based energy structure, and contribute to controlling haze pollution. Therefore, the following hypothesis is further proposed based on the analysis above:

**Hypothesis** **3.** *Industrial co-agglomeration will not only directly affect haze pollution, but also affect it through urbanization and energy structure. We expect that urbanization and energy structure can play a mediating role between industrial agglomeration and haze pollution*.

## 3. Establishment of Empirical Model and Data Explanation

### 3.1. Measurement Model Setting

#### 3.1.1. Spatial Econometric Model

Considering the pollutant spatial diffusion, sources of haze pollution in a region can be divided into three parts:$PM_{it}=E_{it}+S_{jt}-S_{it}$, where *t* is the year; *i* is the cross-section unit; *PM* is the observed value of haze pollution; *E_i_* is the actual generation of haze pollution in region *i*; *S_j_* is the diffusion amount of local haze pollution from other regions; *S_i_* is the part of local pollution spreading to other regions, so it has no effect on the actual emission of local haze; *E_i_*, *S_j_*, and *S_i_* are non-observed values. According to the theory of spatial econometrics, *S_jt_−S_it_* reflects the cross-region spatial dependence of haze pollution, that is, the “contribution” of other regions to local haze pollution. From a static point of view, it is presented as the spatial dependence in the current period ($S_{jt}-S_{it}=\rho \sum_{j}w_{ij}PM_{jt}$) or spatial error characteristic ($S_{jt}-S_{it}=\mu_{it}=\lambda \rho \sum_{j}w_{ij}\mu_{it}+\varepsilon_{it}$). The dynamic spatial panel model constructed by Elhost [52] shows that the spatial dependence of variables can not only be reflected in the current inter-regional correlation, but can also be affected by the previous behavior between regions. In view of this, the spatial dependence and spatial error characteristics of haze pollution are further modified as follows:

(1) $$S_{jt}-S_{it}=\rho \sum_{j}w_{ij}PM_{jt}+\gamma \sum_{j}w_{ij}PM_{j,t-1}\mathrm{and}\;S_{jt}-S_{it}=\xi \mu_{t-1}+\lambda \sum_{j}w_{ij}+\varepsilon_{it}$$

To test Hypothesis 1, we introduce the square term of industrial co-agglomeration (*sAgco*) into the model. The observation of haze pollution can be expressed as follows:

(2) $$PM_{it}=\alpha_{0}Agco_{it}+\alpha_{1}sA{gco}_{it}+\alpha_{2}X_{it}+\rho \sum_{j}w_{ij}PM_{jt}+\gamma {\sum_{j}PM}_{j,t-1}+u_{it}$$

(3) $$PM_{it}=\beta_{0}A{\mathrm{gco}}_{it}+\beta_{1}sAgco_{it}+\beta_{2}X_{it}+\xi \mu_{t-1}+\lambda \sum_{j}w_{ij}\mu_{it}+\phi_{it}$$

where *α* and *β* are coefficient vectors; *X* refers to the vector of control variables; $\phi_{it}=\varepsilon_{it}+u_{it}$, *ε_it_* and *u_it_* are the perturbation terms with normal distribution; *λ* is the coefficient of the spatial error term; *ρ* and *γ* are the current spatial lag coefficient and spatiotemporal lag effect coefficients, respectively, reflecting the haze pollution influence of local regions on surrounding regions, in the current period and lag phase, respectively.

Equations (2) and (3) implicitly assume that haze pollution will change with local factors; in other words, time lag effect is absent in the assumption. However, in reality, macroeconomic variables often have path-dependent characteristics and the previous level has a significant impact on the current results [53]. According to agglomeration externality theory [54], industrial co-agglomeration (*Agco*) has an obvious spatial lag effect, resulting in the expected change of haze pollution also lagging behind [15]. Therefore, it is crucial to investigate the spatial lag effect of haze pollution. Based on this, referring to Shao et al. [53], we transform Equations (2) and (3) into:

(4) $$PM_{it}=\theta PM_{i,t-1}+\alpha_{0}Agco_{it}+\alpha_{1}sAgco_{it}+\alpha_{2}X_{it}+\rho \sum_{j}w_{ij}PM_{jt}+\gamma {\sum_{j}PM}_{j,t-1}+u_{it}$$

(5) $$PM_{it}=\theta^{\prime }PM_{i,t-1}+\beta_{0}Agco_{it}+\beta_{1}sAgco_{it}+\beta_{2}X_{it}+\xi \mu_{t-1}+\lambda \sum_{j}w_{ij}\mu_{it}+\phi_{it}$$

where θ(θ′) is the spatial lag coefficient, reflecting the impact of haze pollution from the previous period on the current period, that is, the intensity of the time lag effect. Equations (4) and (5) are the spatial lag model (SAR) and spatial error model (SEM) of the dynamic spatial panel used in this study. The dynamic spatial panel can comprehensively reflect the time lag, spatial lag, and spatiotemporal lag effects of haze pollution from the single dimension of space-time, and the two dimensions of space and time, making it helpful to obtain more robust estimation results.

#### 3.1.2. Spatial Weight Matrix

Under the influence of economic activities, such as industrial transfer, and natural conditions especially the atmosphere, obvious spatial correlation is found in haze pollution. Consequently, the weight matrix which reflects spatial relationship should be taken into account to study haze pollution. Hence, a spatial weight matrix (W1) of geographical distance is constructed according to the geographical distance between cities to reflect the spatial relation between haze pollution, with geographical factors. Additionally, the economic geography nested matrix (W2) is obtained by point multiplication in MATLAB to reveal geographic and urban economic information of haze pollution, which is applied in robustness tests.

### 3.2. Selection of Variables

#### 3.2.1. Explained Variable: PM_2.5_ (*PM*)

**Figure 2:**
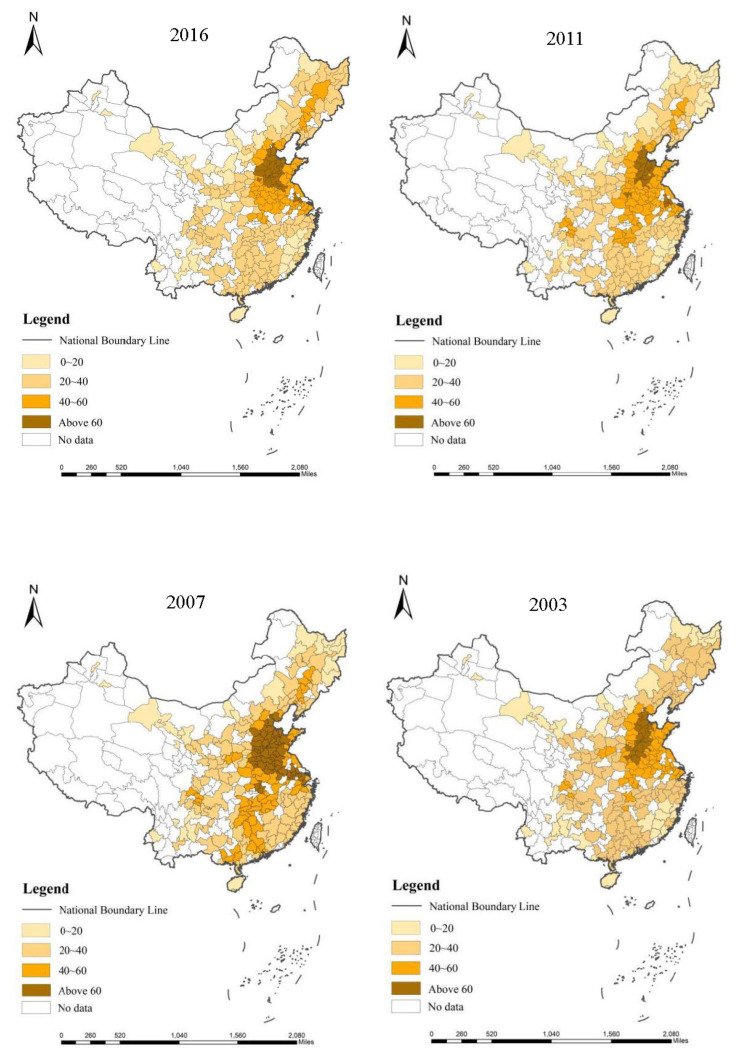
Annual average concentration of PM_2.5_ in Chinese cities from 2003 to 2016.

Obviously, PM_2.5_ is harmful to the atmospheric environment and residents’ health [55]. This study uses the grid data concerning average annual global PM_2.5_ concentration that are jointly released by the Application Center and Social Economic Data of Columbia University based on satellite monitoring [56]. These grid data were further transformed into the annual average PM_2.5_ concentration data of 283 Chinese cities in 2003–2016 by ArcGIS software. The annual average PM_2.5_ concentration in these Chinese cities is divided into four levels by the ArcGIS natural fracture method. Figure 2 shows the distribution of their annual average PM_2.5_ concentration in 2016 to 2003. The darker the color, the higher the concentration of PM_2.5_ and the more serious the haze pollution (Figure 2).

From the comparison of different years, it can be found that from 2003 to 2007, haze pollution has been deteriorating in China, showing a tendency of moving from eastern cities to the middle part of China. From 2007 to 2011, haze pollution has improved in most Chinese regions. In 2011–2016, although areas with severe haze pollution spread, the overall increase of haze pollution slowed down. Moreover, the distribution of haze pollution is not uniform across the country of China, showing the characteristics of continuous distribution. The cities where haze pollution is at the lowest level are primarily located in southern coastal and southwest China, while those suffer from the most serious influence of haze pollution are located on the middle reaches of the Yellow River and the northern coast. In general, haze pollution is decreasing in the east, middle, and western parts of the country. In terms of haze pollution, a high degree of spatial correlation has been observed in each region and there is a phenomenon of pollutant diffusion among regions. This coincides with the gradual transfer of Chinese manufacturing industry from the eastern to western and middle regions [57]. In this context, the spatial econometric model is used in this study to investigate haze pollution in China and explore how industrial co-agglomeration affects haze pollution.

#### 3.2.2. Core Explanatory Variable: Industrial Co-Agglomeration (Agco)

The concept of industrial co-agglomeration was proposed first by Ellison and Glaeser [58], as a special form of industrial agglomeration in the dynamic development process with capital, talent, technology, and information [59], and it is necessary to measure the co-agglomeration between manufacturing and producer services from the perspective of industrial agglomeration. Based on the idea of collaborative agglomeration between industries put forward by Ellison et al. [60] and the practical application of Li [61], this study constructs the co-agglomeration index through location entropy. Furthermore, based on the differences in economic activity agglomeration indicators, the characteristics of co-agglomeration between producer services and manufacturing are described as follows:

(6) $$LQ_{it}=\frac{X_{it}/\sum X_{it}}{Q_{it}/\sum Q_{it}}$$

where *LQ_it_* is the location entropy of manufacturing (producer services) of city *i* in year *t*; *X_it_* refers to quantity employees in the manufacturing (producer services) of city *i* in year *t*; and *Q_it_* is quantity total employees of city *i* in year *t*:

(7) $$LQAgco=\left(1-\frac{\left|LQman-LQser\right|}{LQman+LQser}\right)+|LQman+LQser|$$

In Equation (7), *LQman* is the index of manufacturing agglomeration, *LQser* is the index of producer services agglomeration, and *LQAgco* is the index industrial co-agglomeration. The quality of the co-agglomeration index is represented by the first term on the formula’s right side; the depth of that is represented by the second term; and the sum of the two items represents the degree to which producer services and manufacturing are co-agglomerated. The larger the co-agglomeration index, the higher the degree of co-agglomeration between the two industries. Based on data availability, the producer services in this study involve technical services, R&D design, financial services, information services, postal express services, warehousing services, goods transportation, business services, productive leasing services, environmental protection services, energy conservation services, wholesale and trade brokerage services, vocational education and training services, human resource management and producer support services.

#### 3.2.3. Control Variables

As the STIRPAT model [62] and EKC [63] hypothesis are the basic theoretical frameworks for analyzing environmental pollution research, we combined these two to select the control variables of industrial co-agglomeration on haze pollution from the perspectives of economy, society, resources, and environment. First, for transportation (*Tra*), we measure the traffic intensity of a city by road kilometers. Automobile exhaust emissions from high-intensity road transportation is a major source of haze pollution [64]. Second, for electricity consumption (*Elec*), we use the total social electricity consumption to measure the power consumption scale of the city. Approximately two-thirds of the world’s electricity comes from the burning of fossil energy, with coal as the main fuel, which is also an important source of haze pollution [65]. Third, for consumption level (*Con*), we use the proportion of total retail goods and GDP to represent consumption levels of urban residents [66]. Cities with larger consumption tend to have more production activities, which may negatively affect a number of ecological conditions and further aggravate haze pollution. Fourth, for openness (*Open*), we use foreign direct investment representing the level of opening up. At the same time, the “pollution halo” hypothesis holds that foreign direct investments (*FDI*) can be introduced through green technology to reduce the negative externalities of economic development on environmental pollution [67]. The “pollution haven” hypothesis holds that FDI drives the enhancement of environmental pollution by relocating the highly polluting industries from developed countries [68]. Fifth, for technical level (*Tec*), we use three patent applications to represent the technical level of the city. Higher technical level helps to improve energy utilization efficiency, promote the “green” adjustment of energy structure, and consequently, reduce pollution emissions [69]. Sixth, for GDP per capita (*Eco*), we investigate the association between haze pollution and the growth of urban wealth through the per capita wealth of a city represented by GDP per capita. Seventh, for population density (*Pop*), we use urban population density or the quantity of individuals per unit area, to characterize the environmental impact of urban population agglomeration. The concentration of population and production activities usually aggravates haze pollution [70].

#### 3.2.4. Mediating Variables

The following mediating variables were adopted in this study:

Urbanization (*City*). The ratio of the population in cities to the entire population in the country is usually used to measure urbanization. The rapid development of urbanization may lead to rapid economic development, but will aggravate haze pollution since it usually sacrifices the ecological environment. At the same time, with the promotion of urbanization and improvement of income level, urbanization will produce positive externalities to reduce environmental damage through scale economies, agglomeration, and resource reallocation effect [71].

Energy structure (*Es*). We characterize the energy structure with the ratio of coal consumption to total consumption of energy. In China, coal is the major energy source and the main cause of its environmental pollution problems, which may increase haze pollution [64].

### 3.3. Data Source

The description and data source of each variable are shown in Table 1 based on the availability of data.

## 4. Empirical Regression Results and Discussion

### 4.1. Impact of Industrial Co-Agglomeration on Haze Pollution

#### 4.1.1. Regression Results and Discussion

Before the model estimations, the LM test was used to select spatial error and spatial lag models in accordance with the following criteria. First of all, the model with more significant LM statistics is the more desirable model; if there is a same significance level between two models in terms of the LM statistics, then the setting form of the model needs to be determined by the significance of the robustness of LM statistics [72].

Table 2 illustrates that the robust LM test value for the spatial lag model is significant at the 1% level with the setting of W1 and W2. However, the spatial error model does not have a significant robust LM test value, which indicates that the spatial error model (SEM) is inferior to the spatial lag model (SAR). Therefore, in the following section, we only report and discuss the estimation results of the dynamic spatial lag model based on Formula (4). For comparison, we also report the estimation results under non-spatial panel OLE-FE, non-spatial dynamic panel GMM, and static SAR model in Table 3.

It can be seen from Table 3 that the coefficients of *Agco* and *sAgco* in Models 1 and 3, without considering the time lag of haze pollution, are not significant. However, the estimated coefficient of *Agco* in Model 2, without considering the spatial correlation, is not significant. This indicates that the estimation results without considering the time–space correlation of the explained variables may lead to errors. To obtain more robust estimation results, consideration of the temporal and spatial correlation of haze pollution is indispensable. The estimation results of Model 4, which considered the temporal and spatial correlation of haze pollution, showed better statistical characteristics. It can be observed that, compared to the above three models, the results of Model 4 in which the time lag effect and spatial correlation of haze pollution are taken into consideration have the best measurement performance and theoretical expectation. Therefore, the following discussion focuses on the dynamic spatial panel regression results based on the SAR model.

From the perspective of spatial dimension, the spatial lag coefficient rho is significantly positive at 1% with both weight matrix settings. It proved that there are obvious spatial clustering characteristics as well as a significant spatial spillover effect in terms of urban haze pollution in China. This indicates that under the driving forces caused by weather factors including rainfall temperature difference, wind direction, and other social and economic activities, such as commodity trade, industrial transfer, the level of local haze pollution shows a close relationship with neighboring areas. From the perspective of time dimension, the time lag of haze pollution in W1 and W2 is at 1% significance level. This indicates that the characteristics of path dependence are found in the change of haze pollution. If haze pollution is at a higher level in the current period, the next phase of the haze pollution levels will increase continuously, exhibiting the “snowball effect.”

Under the spatial weight of W1, the coefficient of *Agco* is significantly positive while *sAgco* is significantly negative. This indicates that there is a typical inverted U-shaped curve relationship between industrial co-agglomeration and haze pollution. Hence, Hypothesis 1 is verified. When the industrial co-agglomeration index is lower than 3.6531, it can significantly promote the deterioration of haze pollution. This is largely due to that, in the initial stage of industrial co-agglomeration, producer services and manufacturing industries tend to be concentrated in a single spatial dimension, and the manufacturing industry accounts for a high proportion haze pollution. The resource allocation of production factors has not yet reached the optimal level, which leads to an increase in the cost of environmental pollution management and thus aggravates environmental pollution. When the industrial co-agglomeration index is higher than 3.6531, it can inhibit the deterioration of haze pollution. The reason is that the higher-level of industrial co-agglomeration brings the spillover effect between the two industries on knowledge and green technology. The development of producer services is effectively promoted by manufacturing agglomeration and the output of producer services agglomeration promotes the progress of manufacturing by manpower and knowledge capital in turn. It can add a manufacturing value chain and cut the transaction cost between enterprises. This will help to increase the production efficiency and management level of the manufacturing industry significantly, which is beneficial for haze pollution governance. Based on samples’ observations, the majority of Chinese cities did not cross the inflection point value in 2016, and they would still face greater pressure of haze pollution in the future. Only Shenzhen, Zhuhai, Shanghai, Suzhou, Guangzhou, and Tianjin were above the inflection point. This shows that producer services provide more specialized intermediate input services for manufacturing enterprises through industrial specialization and efficient integration of resources. Furthermore, the scale economic effect of industrial agglomeration has been deepened, promoting innovation and clean production technology in the manufacturing industry; it affects haze governance in a positive pattern.

From the perspective of control variables, with the space weight of W1, the estimated coefficient of transportation on haze pollution is significantly positive, showing that haze pollution is aggravated as a result of the expansion of urban transportation scale. This indicates that transportation is still dominated by traditional vehicles, the application scope of new energy vehicles is limited, and the green travel mode of urban residents needs to be popularized further. The significantly positive coefficient of electricity consumption to haze pollution indicates that China’s power system is mainly based on thermal power generation, and the large-scale use of fossil fuels aggravates haze pollution in cities. In addition, China still receives a lot of high-polluting industries from abroad, which can be seen from the significantly positive estimated coefficient of openness on haze pollution. Under the pressure of performance evaluation, local governments ignore the “green” requirements of FDI while pursuing the scale of FDI, which is harmful to the governance of haze pollution. GDP per capita has a positive but non-significant impact on haze pollution. This indicates that regardless of the economic development of Chinese cities, they should further improve their pollution control level and adopt stricter environmental regulation standards to force enterprises to develop in a green way. Haze pollution is under significant inhibitory influence of the technological level. The improvement of the urban technological level can help improve the green technologies of energy conservation and emission reduction, which would contribute to the reduction of haze pollution and optimize resource utilization. The impact of population density on haze pollution is found to be significantly positive; a higher population density results in more demand for housing, transportation, and energy, and spatial agglomeration of economic activities, which aggravates haze pollution. At the same time, the full combustion of fuel would be negatively affected due to heavy traffic and wind speed is affected by higher residential density. Consequently, it is more difficult for air pollutants to diffuse, thus exacerbating haze pollution in an indirect manner.

#### 4.1.2. Robustness Analysis

This study adopts the replacement of the spatial weight matrix, lag period, and space model for the purpose of testing the robustness of the results above. The following steps are taken to complete this task. Firstly, economic distance nested weight matrix (W2) is used to replace geographical distance spatial weight matrix (W1) for the robustness test. Following that, re-estimations based on the regression are carried out with the highest second-order spatial lag term of the explanatory variable, replacing the first-order spatial lag term used previously. Lastly, based on the (generalized spatial two-stage least squares) GS2SLS model, the spatial lag term is adopted as the tool variable and W1 is used to re-estimate the model. Table 4 gives the test results. The global Moran’s I index reported by GS2SLS and the spatial lag term of haze pollution above the three ways are still significant. There is still an inverted U-shaped curve relationship between *Agco* and *PM*. Therefore, the regression of Model 4 in Table 3 has strong robustness, which verifies the validity of Hypothesis 1 once again.

#### 4.1.3. Direct and Indirect Effects

With the presence of spatial spillover effect, a change in an influencing factor will cause a change in local haze pollution, and produces an impact on the corresponding neighboring areas, which will cause various adjustment changes through the cyclic feedback effect. Accordingly, there are direct and indirect effects on haze pollution when various factors are taken into account. The overall impact of a certain factor change on haze pollution in this region is a direct effect. An indirect effect is reflected in the influence of a certain factor on haze pollution in other regions, which is exerted through the spatial spillover effect [73]. This study adopts the dynamic spatial panel data model according to Formula (4). In terms of time dimension, these effects are also divided into long- and short-term effects [4], which, respectively, reflect the direct and indirect effect of a factor on haze pollution in the short and long run. The calculation formula for the above effects is as follows:

(8) $$SR-Direct={\left[{\left(I-\rho W\right)}^{-1}(\alpha_{1k}I_{N})\right]}^{d}$$

(9) $$SR-Indirect={\left[{\left(I-\rho W\right)}^{-1}(\alpha_{1k}I_{N})\right]}^{\bar{rsum}}$$

(10) $$LR-Direct={\left\{\left[(1-\theta )I-{\left((\rho +\gamma )W\right)}^{-1}(\alpha_{1k}I_{N})\right]\right\}}^{d}$$

(11) $$LR-Indirect={\left\{\left[(1-\theta )I-{\left((\rho +\gamma )W\right)}^{-1}(\alpha_{1k}I_{N})\right]\right\}}^{\bar{rsum}}$$

*I* is a unit matrix; *d* represents the operator which calculates the mean of diagonal elements in the matrix; *rsum* represents the operator which calculates the row and average of non-diagonal elements in the matrix; other variables have the same meaning as above.

Table 5 shows the decomposition results of the influence effects based on the results of Table 3 and Model 4. Two types of spatial weight matrix can be found, the direction of *Agco* is positive and *sAgco* is negative. The long-term effects of the absolute influence degree coefficient (absolute value) are greater than the short-term, indicating that industry co-agglomeration has a more profound influence on haze pollution in the long term. Taking the discussion of W1 as an example, this study focuses on industrial co-agglomeration, which is its core explanatory variable. Regardless of the short- and long-term conditions, the impact of the core explanatory variable on the explained variable is positive (0.0087 and 0.0272, respectively), but the indirect effect is negative. This indicates that industrial co-agglomeration has a promoting impact on local haze pollution as well as an inhibiting effect in neighboring areas. Hence, Hypothesis 2 was verified. In the beginning, many traditional manufacturing industries clustered in the region, resulting in an increase in demand for production factors and public facilities. It will further expand production and aggravate local haze pollution. Meanwhile, a large number of workers and high-polluting industries move out of neighboring areas to pursue higher economic benefits, whereas the haze pollution in neighboring areas is alleviated. This result can be explained by the warning effect: high haze pollution caused by a large number of production activities may lead to an increase in the demands of local and surrounding habitants for environmental governance. This increased the governments’ attention in surrounding areas concerning the strengthening of environmental regulations and the control of haze pollution, which is conducive to “haze reduction” [4]. The *sAgco* affects local haze pollution negatively and has a positive impact on surrounding areas. For this reason, when the industry co-agglomeration level reaches a certain extent, it results in the reduced pollution from advanced manufacturing industry and producer services collaborative agglomeration. Meanwhile, high-pollution industries will face elimination or transfer from the neighborhood, which will inhibit local haze pollution and promote surrounding haze pollution.

As for control variables, in both the short and long term, population density, consumption level, and the scale of transportation affect local haze pollution positively, but have a negative spillover effect on the surroundings. This is because the areas more advanced traffic networks, higher consumption levels, and higher population densities, tend to be more developed. These areas are often surrounded by regions of high population and industry has a certain siphon effect, which causes increase in local haze pollution and reduction in surrounding regions. The scale of electricity consumption and openness increases haze pollution in the surrounding areas while aggravating local haze pollution. The improved technological level is beneficial for the reduction of local haze pollution. Although the transfer of clean technology between regions has a certain time lag, it cannot significantly inhibit surrounding haze pollution. As shown in Table 4 (Model 4), the coefficient of GDP per capita is not significant, so that its direct and indirect effects are also not statistically significant.

#### 4.1.4. Regional Sample Regression

In this study, we divided China’s territory into three parts (eastern, central, and western) to verify the differences in industrial co-agglomeration of different regions on haze pollution. Table 6 reports the estimation results of integrating the geographical distance spatial weight matrix (W1) into the dynamic spatial model.

Table 6 shows that in terms of the time-space lag coefficient of the explained variable, the positive spillover effect of haze pollution is the strongest in eastern, followed by central and western China, all significant at 1%. The reason lies that there is a strong spatial correlation in eastern and central China in the aspect of energy consumption, industrial transfer, and population mobility. In contrast, the western region has a weak industrial base and a relatively slow process of production activities and there is a small promoting spillover effect of its haze pollution. From the perspective of industrial co-agglomeration and haze pollution, there is a significant inverted U-shaped curve relationship in central and eastern China. It means that with the industrial co-agglomeration level increases, haze pollution in the eastern and central regions first shows an upward trend and then by a downward trend. However, the relation between industrial co-agglomeration and haze pollution in western China stays at the left part of the curve; that is, haze pollution is aggravated due to the improved level of industrial co-agglomeration. In terms of the regression coefficient between *Agco* and *PM*, haze pollution in the central region receives the strongest promoting effect, whereas the eastern region has the greatest restraining effect. From the point of control variables, for China’s eastern and central cities, transportation scale, electricity consumption, consumption level, openness, and the increase of population density are not conducive to manage the regional haze pollution; only the technical level to haze pollution showed significant inhibitory effect. Furthermore, only transport and electricity consumption showed a significant promoting effect for haze pollution. First, the eastern region has a good industrial foundation and the technology and knowledge-intensive manufacturing industries occupy a relatively high rate. With the improvement in the level of industrial co-agglomeration, producer services are more and more critical in supporting the manufacturing industry, promoting its green development, which helps alleviate haze pollution. Moreover, there is a higher level of economic development in the eastern region and the public exerts a stronger desire for the amelioration of the ecological environment. The government will adopt stricter environmental regulation policies to develop green industries and screen “environment-friendly” FDI to slow down the damage caused by economic development to the ecological environment. However, under the unbalanced regional economic development pattern, in the process of industrialization, some cities in the western region adopt an extensive economic growth mode, accepting the polluting industries eliminated from the eastern region. Objectively, this will cause haze pollution to increase in the central and western regions.

### 4.2. Mediating Effect Tests Based on Urbanization and Energy Structure

According to Hypothesis 3, industrial co-agglomeration may influence haze pollution through urbanization and energy structure. To test whether urbanization and energy structure act as mediating variables, this study adopts a standardized mediating effect model [74] and conducts further empirical investigation based on the spatial lag model. The specific mediation effect test model was set as follows:

(12) $$M_{it}=\theta M_{i,t-1}+\pi_{0}Agco_{it}+\pi_{1}sAgco_{it}+\alpha_{2}X_{it}+\rho \sum_{j}w_{ij}PM_{jt}+\gamma {\sum_{j}PM}_{j,t-1}+u_{it}$$

(13) $$\begin{array}{l} PM_{it}=\theta PM_{i,t-1}+\omega_{0}Agco_{it}+\omega_{1}sAgco_{it}+\eta M_{it}+\alpha_{2}X_{it}+\\ \rho \sum_{j}w_{ij}PM_{jt}+\gamma {\sum_{j}PM}_{j,t-1}+u_{it} \end{array}$$

where *M* is the possible mediating variable. Equations (4), (12), and (13) form a complete mediating effect test. If the coefficients of industrial co-agglomeration *ω*_0_ and *ω*_1_, *π*_0_ and *π*_1_, and *η* are significant, and *ω*_0_ and *ω*_1_ are smaller or significantly lower than those of *α*_0_ and *α*_1_, the mediating effect exists.

Table 7 reports the intermediary effect estimation results when urbanization is an intermediary variable. The term coefficients of *Agco* and *sAgco* in Equations (4) and (12) are significantly, the coefficients in Equation (13) compared with Equation (4) are lower. Thus, urbanization is the intermediary variable between industrial co-agglomeration and haze pollution. That is, industrial co-agglomeration can significantly promote the level of urbanization and affect haze pollution. Urbanization significantly promotes the deterioration of haze pollution, indicating that the improvement of urbanization leads to the rapid accumulation of people and wealth in cities, which is not conducive to the treatment of haze pollution. In the process of urbanization, most cities blindly pursue the expansion of scale and the demand for infrastructure construction continues to expand, which drives the excessive growth of high-pollution industries dominated by cement and steel, resulting in the increase of haze pollution. Apart from that, a lot of dust has been produced during the construction of urban infrastructure, which objectively increases haze pollution. For the energy structure, the term coefficients of *Agco* and *sAgco* in Equations (4) and (12) were significantly and the coefficients in Equation (13) were lower than those in Equation (4). Based on this, it can be concluded that the energy structure is an intermediary variable that affects haze pollution. Industrial co-agglomeration which increases the proportion of coal in the energy structure has an impact on haze pollution. This shows that haze pollution is aggravated as a result of an increase in the coal consumption proportion. The proportion of coal consumption in China has been maintained at approximately 70% for a long time. This coal-based energy structure has undoubtedly become the main culprit for atmospheric environmental pollution represented by haze pollution.

Therefore, the following conclusions can be drawn: industrial co-agglomeration increases haze pollution through urbanization and energy structure; hence, Hypothesis 3 is valid. In addition, the coefficients of *Agco* and *sAgco* are positive and negative, respectively, indicating that industrial co-agglomeration will eventually restrain haze pollution. At the same time, more efforts should be made in the process of urbanization to strictly control the inefficient expansion of urban scale and to maximize the restraining effect of industrial coordinated agglomeration by reducing the proportion of coal in energy consumption. Moreover, in the process of urbanization, we should also pay attention to strictly controlling the inefficient expansion of urban scale and cut down the ratio of coal consumption in the energy structure to maximize the inhibitory effect of industrial co-agglomeration on haze pollution.

## 5. Conclusions

Haze pollution has a strong spatiotemporal dependence, that is, haze pollution shows path dependence in time dimension and also has significant spatial correlation. If haze pollution in the region was at a high level in the current period, in the next phase it may continue to rise. In terms of spatial dimension, haze pollution among regions showed significant positive spatial correlation, mainly concentrated in the northern coastal areas of China and the middle reaches of the Yellow River. It indicates that the haze control efforts of a single city may become futile because of the “leakage effect” among cities. Moreover, the space-time correlation characteristics of haze pollution will also cause damage to residents’ health and lead to public health crisis. Therefore, haze control cannot rely on a single city, which urges the need for close cooperation between cities in the region. Government should clarify the responsibilities of pollution control in different areas, further building a unified environmental pollution monitoring platform, and implementing regional environmental information sharing.

There is a significant inverted U-shaped curve relationship between industrial co-agglomeration and haze pollution in China. Only when the industrial co-agglomeration index is higher than 3.6531, it will have a significant inhibitory effect on haze pollution. Most Chinese cities are located below the inflection point, which shows that “haze control” will be a hard, long-term task. Therefore, the government must introduce more active environmental regulation policies and measures as soon as possible to realize the decoupling of economic growth and haze pollution. Whether it is a long or short-term effect, when the industrial co-agglomeration index is lower than 3.6531, it can promote haze pollution in local regions but inhibit it in surrounding areas. When the industrial co-agglomeration index is higher than the inflection point, it is helpful for haze governance in local region but has a negative impact in neighboring areas. It shows that each region needs to unify the environmental regulation action, forming the regional joint force of haze pollution control. Therefore, in order to realize effective control of haze pollution, we should continuously improving the level of urban industrial co-agglomeration, but also reduce the proportion of high pollution and consumption manufacturing industry, develop producer services with high-added-value and low-pollution vigorously. More importantly, the government should also formulate the market-oriented environmental regulations to force the green upgrading of industrial structure.

The results of regional regression show the existence of a typical inverted U-shaped curve correlation between industrial co-agglomeration and haze pollution across China, including central and eastern regions. Industrial co-agglomeration first acts as a resistance to environmental pollution control. But with the deepening of co-agglomeration, this influence gradually transforms into a positive role. However, industrial agglomeration in Western China can only contribute to worse haze pollution. Therefore, local conditions should also be taken into account when formulating the policy of co-agglomeration of producer services and manufacturing industries. Specifically, the eastern region has a good industrial foundation and the manufacturing industry with its knowledge and technology-intensity accounts for a large proportion. We should further support the development of producer services and transform the tacit technology and knowledge into the manufacturing productivity. Due to the high proportion of labor-intensive low-end manufacturing industries and the lack of supporting producer services in the central and western regions, we should vigorously introduce and cultivate relevant supporting producer services, pay attention to the horizontal and vertical expansion of the industrial chain, and improve the level of industrial co-agglomeration to enhance the inhibitory effect on haze pollution.

Besides, improved industrial co-agglomeration levels can work through urbanization and energy structure and produce an indirect impact on haze pollution. In other words, urbanization and energy structure are the transmission channels for industrial co-agglomeration to exert its effect on haze pollution. Hence, related measures should be taken to improve the utilization efficiency of urban land resources, build efficient and convenient urban transportation infrastructure, and promote the intensive development of the city. Moreover, it is necessary to increase support for research and development of technologies concerning pollution prevention and energy conservation, and formulate appropriate fiscal and tax preferential policies that can motivate companies to engage in green technology innovation activities. Finally, we should vigorously promote the application of green energy, continue to reduce the proportion of oil, coal and other traditional energy resources, accelerate the market-oriented reform of energy prices, and realize the gradual replacement of traditional energy by green and clean energy relying on a market-oriented mechanism.

With the development of China’s economy, environmental issues, especially air pollution, have aroused widespread concern. It not only brings inconvenience to residents’ daily life, but also awakens the public’s awareness of environmental protection. This paper uses PM_2.5_ concentration data monitored by satellite to more accurately reflect the spatiotemporal evolution of haze pollution in China, and improves the theoretical mechanism of industrial agglomeration on haze pollution from the perspective of co-agglomeration between manufacturing and producer services, aiming to relieve the contradiction of industrial development and environmental pollution in China, and provide a new management practice for sustainable development of economy. This research method can also be applied to study the impact mechanism of single industrial agglomeration on environment pollution, and identify the influencing factors of China’s environmental pollution, and provide economic policies for China’s pollution control. Due to the incomplete industrial categories of producer services and manufacturing in some cities of China, the co-agglomeration index at city level can only reflect the relative state of industrial co-agglomeration. The data of segmented industries should be used to calculate the co-agglomeration index according to the existing industrial categories of cities, so as to more truly reflect the level of urban industrial co-agglomeration from a micro perspective. Because of the cross-regional flow of talents, capital, technology, and other factors as well as industries, co-agglomeration will also occur among cities. Therefore, the impact of cross-city producer services and manufacturing on haze pollution needs to be further expanded.

## Figures and Tables

**Table 1:** Data Selection & Description.

Variable Type	Index	Sign	Description	Source
Explanatory variable	Haze Pollution	*PM*	Annual average concentration of PM_2.5_	(https://beta.sedac.ciesin.columbia.edu/)
Core explanatory variable	Industrial Co-agglomeration	*Agco*	Industrial co-agglomeration between manufacturing and producer services	Calculated by Equations (7) and (8)
Control variable	Transportation	Tra	Urban road area (Km^2^)	China City Statistical Yearbook (2004–2017)
	Electricity consumption	*Elec*	Total electricity consumption of the whole society (Kw/h)	
	Consumption level	*Con*	Total retail goods (10^4^ yuan)/GDP	
	Openness	*Open*	FDI (10^4^ yuan)	
	Technical level	*Tec*	Number of patent applications(pieces)	
	GDP per capita	*Eco*	GDP/population	
	Population density	*Pop*	Population/regional administrative area	
Mediating variable	Urbanization	*City*	Urban population/total population	
	Energy structure	*Es*	Total coal consumption/total energy consumption	

**Table 2:** LM test of spatial panel model.

Test Project	W1		W2	
	χ2	*p*-Value	χ2	*p*-Value
LM-lag	127.91	0.000	128.95	0.000
LM-error	134.88	0.000	134.82	0.000
LM-lag (Robust)	7.455	0.000	7.940	0.005
LM-error (Robust)	1.955	0.157	1.498	0.229

**Table 3:** The impact of industrial co-agglomeration on haze pollution.

Variable	OLS-FE	GMM	SAR	Dynamic SAR
	Model 1	Model 2	Model 3	Model 4
L.LnPM		0.6564 *** (23.39)		0.6853 *** (19.27)
LnAgco	0.0024 (0.61)	0.0039 * (1.92)	0.0062 (0.19)	0.0089 *** (2.78)
LnsAgco	−0.0001 (0.43)	−0.0002 (1.60)	−0.0001 (−0.17)	−0.0004 ** (−2.17)
InTra	0.0135 *** (2.77)	0.0161 *** (4.13)	0.0079 * (1.91)	0.1542 *** (2.88)
InElec	0.0056 ** (2.21)	0.0044 ** (2.03)	0.0047 ** (2.20)	0.0220 *** (2.93)
InCon	0.0056 ** (2.16)	0.0105 *** (3.35)	0.0131 ** (2.33)	0.0368 *** (5.20)
InOpen	0.0074 *** (2.96)	0.0073 *** (3.01)	0.0035 * (1.74)	0.0075 *** (3.52)
InTec	−0.0014 * (−1.92)	−0.0130 ** (−2.52)	−0.0087 * (−1.90)	−0.0217 *** (−3.50)
InEco	−0.0124 (−1.58)	−0.0128 *** (−1.28)	−0.0135 ** (−2.11)	−0.0152 (−0.23)
InPop	0.0162 (0.91)	0.0723 *** (9.48)	0.0260 * (1.79)	0.0345 *** (3.43)
Log			834.4522	819.7080
Rho			0.0105 *** (22.32)	0.5755 *** (30.63)
R^2^	0.4400		0.6424	0.8951
AR (2) [P]		1.182 (0.3222)		
Sargan [P]		143.38 (0.2311)		
Obs	3962	3962	3962	3962

Note: The values in brackets are *T*-value, with ***, ** and * representing the significance level of 1%, 5% and 10% respectively; sAgco is the square term of industrial co-agglomeration; the following tables are the same.

**Table 4:** Robustness check.

Variable	Replace Spatial Weight Matrix	Replace Time-Lag Order	Replace Spatial Model
	W2	Two Periods of Lag	GS2SLS
L.InPM	0.6709 *** (3.94)	0.5036 *** (3.48)	0.6120 *** (6.38)
InAgco	0.0067 *** (2.82)	0.0076 *** (2.77)	0.0053 *** (3.21)
InsAgco	−0.0003 *** (−2.92)	−0.0002 ** (−1.99)	−0.0003 *** (−2.77)
InTra	0.1558 *** (3.58)	0.1310 *** (3.18)	0.1456 *** (3.46)
InElec	0.0230 ** (3.72)	0.0205 *** (3.14)	0.0208 ** (2.00)
InCon	0.0229 ** (2.19)	0.0327 *** (2.97)	0.0233 ** (2.02)
InOpen	0.0089 *** (4.02)	0.0077 *** (3.70)	0.0053 ** (2.36)
InTec	−0.0217 ** (−2.40)	−0.0145 *** (−3.13)	−0.0316 *** (−3.99)
InEco	−0.0155 (−0.33)	−0.0135 (−0.14)	−0.0166 (−0.04)
InPop	0.0355 ** (2.43)	0.0394 *** (2.81)	0.0383 *** (5.33)
Log	878.9064	870.0668	1167.2516
Global Moran’I [P]			0.1116 *** (0.000)
Rho	0.5579 (27.78)	0.9192 *** (48.90)	
R^2^	0.8733	0.6656	0.5152
Obs	3962	3962	3962

Note: The values in brackets are *T*-value, with ***, ** representing the significance level of 1%, 5% respectively.

**Table 5:** Decomposition results of Agco influence on haze pollution under matrix 1 and 2.

W	Effect	InAgco	InsAgco	InTra	InElec	InCons	InOpen	InTec	InEco	InPop
W1	SR-Direct	0.0087	−0.0002	0.0153	0.0252	0.0381	0.0132	−0.0226	0.0013	0.0610
	SR-Indirect	−0.0115	0.0005	−0.0202	0.0333	−0.0503	0.0174	0.0299	−0.0018	−0.0805
	LR-Direct	0.0272	−0.0004	0.0477	0.0787	0.1187	0.0412	−0.0706	0.0044	0.1901
	LR-Indirect	−0.0295	0.0005	−0.0517	0.0853	−0.1287	0.0447	0.0765	−0.0047	−0.2061
W2	SR-Direct	0.0082	−0.0004	0.0160	0.0244	0.0311	0.0094	−00231	0.0025	0.0645
	SR-Indirect	−0.0153	0.0002	−0.0212	0.0599	−0.0347	0.0125	−0.0273	−0.0028	−0.0757
	LR-Direct	0.0241	−0.0004	0.0166	0.0746	0.1105	0.0397	−0.0687	0.0055	0.1773
	LR-Indirect	−0.0290	0.0005	−0.0359	0.0810	−0.1248	0.0411	0.0693	−0.0058	−0.2188

**Table 6:** Regional sample regression.

Variable	Eastern	Central	Western
L.InPM	0.6298 *** (19.29)	0.5875 *** (27.12)	0.2034 *** (7.87)
LnAgco	0.0023 *** (2.64)	0.0192 *** (2.22)	0.0128 * (1.62)
LnsAgco	−0.0007 *** (−3.92)	−0.0004 * (−1.69)	0.0003 (0.36)
InTra	0.1333 ** (1.99)	0.0050 ** (2.18)	0.0032 ** (2.11)
InElec	0.0198 * (1.95)	0.0144 ** (2.14)	0.033 * (1.82)
InCon	0.0028 ** (2.34)	0.0277 * (1.74)	0.0064 (0.71)
InOpen	0.0070 ** (2.26)	0.0074 * (1.79)	0.0032 (0.89)
InTec	−0.0196 *** (−2.68)	−0.0308 *** (−3.83)	−0.0035 (−0.46)
InEco	−0.0005 (−0.08)	−0.0537 (−0.84)	−0.0146 (−1.02)
InPop	0.0058 ** (2.23)	0.0730 *** (2.76)	0.0556 (0.68)
Log	678.9064	670.0668	505.6538
Rho	1.3942 *** (21.20)	1.4459 *** (54.26)	0.8303 *** (23.35)
R^2^	0.7297	0.5928	0.7321
Obs	1313	1300	1066

Note: The values in brackets are *T*-value, with ***, ** and * representing the significance level of 1%, 5% and 10% respectively.

**Table 7:** Mediating Effect Tests Based on Urbanization and Energy Structure.

Variable	M = Urbanization			Variable	M = Energy Structure		
	(4)	(12)	(13)		(4)	(12)	(13)
L.InCity		0.4466 *** (9.38)		L.InEs		0.4943 *** (42.61)	
L.InPM	0.6824 *** (49.57)		0.6814 *** (49.50)	L.InPM	0.6824 *** (49.57)		
InCity			0.0103 ** (2.24)	InEs			0.0300 *** (4.00)
LnAgco	0.0090 *** (2.84)	0.0147 *** (2.80)	0.0076 *** (2.84)	LnAgco	0.0090 *** (2.84)	0.0105 *** (3.23)	0.0057 * (1.81)
LnsAgco	−0.0004 ** (−2.01)	0.0021 ** (2.29)	−0.0003 ** (−1.98)	Ln(Agco)^2^	−0.0004 ** (−2.01)	0.0032 ** (2.21)	−0.0002 ** (−2.03)
Control	Yes	Yes	Yes	Control	Yes	Yes	Yes
Log	834.4522	683.0518	833.1456	Log	834.4522	635.7155	879.0334
Rho	4.1853 *** (20.62)	0.1751 * (1.67)	4.1479 *** (21.78)	Rho	4.1853 *** (20.62)	0.1396 *** (4.33)	3.1629 *** (58.09)
R^2^	0.8688	0.7393	0.8691	R2	0.8688	0.8958	0.9439
Obs	3962	3962	3962	Obs	3962	3962	3962

Note: The values in brackets are *T*-value, with ***, ** and * representing the significance level of 1%, 5% and 10% respectively.

## Data Availability

No new data were created or analyzed in this study. Data sharing is not applicable to this article.

## References

[1] Ke H.Q., Yang W.Y., Liu X.Y., Fan F. (2020). Does Innovation Efficiency Suppress the Ecological Footprint? Empirical Evidence from 280 Chinese Cities. Int. J. Environ. Res. Public Health.

[2] Dong F., Yu B., Pan Y. (2019). Examining the synergistic effect of CO_2_ emissions on PM_2.5_ emissions reduction: Evidence from China. J. Clean. Prod..

[3] Zhao S., Liu S.L., Hou X.Y., Cheng F.Y., Wu X., Dong S.K., Beazley R. (2018). Temporal dynamics of SO2 and NOX pollution and contributions of driving forces in urban areas in China. Environ. Pollut..

[4] Shao S., Li X., Cao J.H., Yang L.L. (2016). China’s Economic Policy Choices for Governing Smog Pollution Based on Spatial Spillover Effects. Econ. Res. J..

[5] Ma L.M., Zhang X. (2014). The Spatial Effect of China’s Haze Pollution and the Impact from Economic Change and Energy Structure. China Ind. Econ..

[6] Liang W., Yang M., Zhang Y.W. (2017). Will the increase of the urbanization rate inevitably exacerbate haze pollution? A discussion of the spatial spillover effects of urbanization and haze pollution. Geogr. Res..

[7] Shen J., Wei Y.D., Yang Z. (2017). The impact of environmental regulations on the location of pollution-intensive industries in China. J. Clean. Prod..

[8] Lin F.Q. (2017). Trade openness and air pollution: City-level empirical evidence from China. China Econ. Rev..

[9] Cai H.Y., Xu Y.Z. (2018). Co-agglomeration, trade openness and haze pollution. China Popul. Resour. Environ..

[10] Amiti M. (2005). Location of vertically linked industries: Agglomeration versus comparative advantage. Eur. Econ. Rev..

[11] Kolko J. (2010). Urbanization, agglomeration and co-agglomeration of service industries. Agglom. Econ..

[12] Gao J.M., Li X.H. (2011). Theoretical and Empirical Study on the Interactive Mechanism between Producer Services and Manufacturing. China Ind. Econ..

[13] Lin B.Q., Tan R.P. (2019). Economic agglomeration and green economy efficiency in China. Econ. Res. J..

[14] Yang H.Z., Tao W., Liu Y., Qiu M.H., Liu J.F., Jiang K.J., Yi K., Xiao Y., Tao S. (2019). The contribution of the Beijing, Tianjin and Hebei region’s iron and steel industry to local air pollution in winter. Environ. Pollut..

[15] Liu S., Fan F., Zhang J.Q. (2019). Are Small Cities More Environmentally Friendly? An Empirical Study from China. Int. J. Environ. Res. Public Health.

[16] Zhang K., Wang D.F. (2014). The interaction and Spatial Spillover between Agglomeration and Pollution. China Ind. Econ..

[17] Li T., Han D., Feng S., Liang L. (2019). Can industrial co-agglomeration between producer services and manufacturing reduce carbon intensity in China?. Sustainability.

[18] Ma J., Wang J., Szmedra P. (2020). Does Environmental Innovation Improve Environmental Productivity?—An Empirical Study Based on the Spatial Panel Data Model of Chinese Urban Agglomerations. Int. J. Environ. Res. Public Health.

[19] Zhao H., Cao X., Ma T. (2020). A spatial econometric empirical research on the impact of industrial agglomeration on haze pollution in China. Air Qual. Atmos. Health.

[20] Lu W., Tam V.W., Du L., Chen H. (2020). Impact of industrial agglomeration on haze pollution: New evidence from Bohai Sea Economic Region in China. J. Clean. Prod..

[21] Liu J., Zhao Y.H., Cheng Z.H., Zhang H.M. (2018). The Effect of Manufacturing Agglomeration on Haze Pollution in China. Int. J. Environ. Res. Public Health..

[22] Chen S.Y., Chen D.K. (2018). Air pollution, Government Regulations and High-quality Economic Development. Econ. Res. J..

[23] Zhang J., Yu H., Zhang K., Zhao L., Fan F. (2021). Can Innovation Agglomeration Reduce Carbon Emissions? Evidence from China. Int. J. Environ. Res. Public Health.

[24] Vallés-Giménez J., Zárate-Marco A. (2020). A Dynamic Spatial Panel of Subnational GHG Emissions: Environmental Effectiveness of Emissions Taxes in Spanish Regions. Sustainability.

[25] Anselin L. (2001). Spatial effects in econometric practice in environmental and resource economics. Am. J. Agric. Econ..

[26] Hong Y., Lyu X., Chen Y., Li W. (2020). Industrial agglomeration externalities, local governments’ competition and environmental pollution: Evidence from Chinese prefecture-level cities. J. Clean. Prod..

[27] Hen N., Peng H. (2020). Can industrial agglomeration achieve the emission-reduction effect?. Socio-Econ. Plan. Sci..

[28] Yan F.Z., Su L., Qiao J. (2011). The exploration of the relationship between the industrial agglomeration’s developments and the environment pollution—the evidence from the manufacturing in China. Stud. Sci. Sci..

[29] Li S.Y., Wang S.J. (2014). An Empirical Study on the Effect of Industrial Agglomeration on Pollution in China. Stat. Decis..

[30] Yoon S., Nadvi K. (2018). Industrial clusters and industrial ecology: Building ‘eco-collective efficiency’ in a South Korean cluster. Geoforum..

[31] Chen C., Sun Y., Lan Q., Jiang F. (2020). Impacts of industrial agglomeration on pollution and ecological efficiency-A spatial econometric analysis based on a big panel dataset of China’s 259 cities. J. Clean. Prod..

[32] Yang Z., Song T., Chahine T. (2016). Spatial representations and policy implications of industrial co-agglomerations, a case study of Beijing. Habitat Int..

[33] Solheim M.C.W., Tveterås R. (2017). Benefitting from co-location? Evidence from the upstream oil and gas industry. Extr. Ind. Soc..

[34] Liu J., Wang J.W., Cheng Z.H. (2017). Research on the influence of Industrial Agglomeration on the Collaborative Innovation Efficiency. China Soft. Sci..

[35] Ding W., Gilli M., Mazzanti M., Nicolli F. (2016). Green inventions and greenhouse gas emission dynamics: A close examination of provincial Italian data. Environ. Econ. Policy Stud..

[36] Tu Z.G. (2014). Strategic Measures to Reduce China’s Carbon Emissions: Based on an Index Decomposition Analysis of Carbon Emissions in Eight Industries. Soc. Sci. China.

[37] Brakman S., Garretsen H., Gigengack R., Van Marrewijk C., Wagenvoort R. (1996). Negative Feedbacks in the Economy and Industrial Location. J. Reg. Sci..

[38] Virkanen J. (1998). Effect of Urbanization on Metal Deposition in the Bay of Toolonlahti, Southern of Finland. Mar. Pollut. Bull..

[39] Ren W.W., Zhong Y., Meligrana J., Anderson B., Watt W.E., Chen J.K., Leung H.K. (2003). Urbanization, Land Use, and Water Quality in Shanghai: 1947–1996. Environ. Int..

[40] Zhang K., Dou J.M. (2013). Study on the Mechanism of Agglomeration on Environmental Pollution. Chin. J. Popul. Sci..

[41] Dong F., Wang Y., Zheng L., Li J.Z., Xie S.X. (2020). Can industrial agglomeration promote pollution agglomeration? Evidence from China. J. Clean. Prod..

[42] Arfi W.B., Hikkerova L., Sahut J.M. (2018). External knowledge sources, green innovation and performance. Technol. Forecast. Soc. Chang..

[43] Zeng D.Z., Zhao L.X. (2009). Pollution havens and industrial agglomeration. J. Econ. Manag..

[44] Ji S.H., Zhu Y.M. (2017). The Study of the Industrial Agglomeration Effect on Misallocation of Resources. J. Quant. Tech. Econ..

[45] Zhang H., Han A.H., Yang Q.L. (2017). Spatial effect analysis of synergetic agglomeration of manufacturing and producer services in China. J. Quant. Tech. Econ..

[46] Shen N. (2014). Spatial agglomeration, scale threshold and technological innovation: An Empirical Analysis Based on census data of Chinese manufacturing enterprises. J. Manag. Eng..

[47] Xiong L., De Jong M., Wang F., Cheng B., Yu C. (2018). Spatial Spillover Effects of Environmental Pollution in China’s Central Plains Urban Agglomeration. Sustainability.

[48] Li X., Xu Y., Yao X. (2021). Effects of industrial agglomeration on haze pollution: A chinese city-level study. Energy Policy.

[49] Fan Q., Yang S., Liu S. (2019). Asymmetrically spatial effects of urban scale and agglomeration on haze pollution in China. Int. J. Environ. Res. Public Health.

[50] Yang Y., Tang D., Yang X. (2020). Investigating the spatio-temporal variations of the impact of urbanization on haze pollution using multiple indicators. Stoch. Environ. Res. Risk Assess..

[51] Song Y., Zhao C., Zhang M. (2019). Does haze pollution promote the consumption of energy-saving appliances in China? An empirical study based on norm activation model. Res. Conserv. Recycl..

[52] Elhorst J.P. (2012). Dynamic Spatial Panels: Models, Methods and Inferences. J. Geogr. Syst..

[53] Shao S., Yang L.L., Huang T. (2013). Theoretical Model and Experience from China of Energy Rebound Effect. Econ. Res. J..

[54] Marshall A. (1982). Elements of the Economics of Industry.

[55] Fan F., Cao D., Ma N. (2020). Is Improvement of Innovation Efficiency Conducive to Haze Governance? Empirical Evidence from 283 Chinese Cities. Int. J. Environ. Res. Public Health.

[56] Donkelaar A.V., Martin R., Brauer M., Boys B. (2015). Use of Satellite Observations for Long-term Exposure Assessment of Global Concentrations of Fine Particulate Matter. Environ. Health Perspect..

[57] Fan F., Lian H., Liu X.Y., Wang X.L. (2021). Can environmental regulation promote urban green innovation Efficiency? An empirical study based on Chinese cities. J. Clean. Prod..

[58] Ellsion G., Glaeser E.L. (1997). Geographic concentration in US manufacturing industries: A dartboard approach. J. Polit. Econ..

[59] Helsley R.W., Strange W.C. (2014). Co-agglomeration clusters, and the scale and composition of cities. J. Polit. Econ..

[60] Ellison G., Glaeser E.L., Kerr W.R. (2007). What Cause Industry Agglomeration? Evidence from Co-Agglomeration Patterns. Am. Econ. Rev..

[61] Li B. (2017). The interaction of clusters between manufacturing and producer services in China. Econ. Res..

[62] Dietz T., Rosa E.A. (1994). Rethinking the environmental impacts of population, affluence and technology. Hum. Ecol. Rev..

[63] Grossman G., Krueger A. (1995). Economic growth and the environment. Q. J. Econ..

[64] Shao S., Zhang K., Dou J.M. (2019). Effects of Economic Agglomeration on Energy Saving and Emission Reduction: Theory and Empirical Evidence from China. Manag World.

[65] Shi Q., Lai X.D. (2013). Identifying the underpin of green and low carbon technology innovation research: A literature review from 1994 to 2010. Technol. Forecast. Soc. Chang..

[66] Wang X.L., Wang S., Fan F., Ye X.H. (2020). Marketization as a channel of international technology diffusion and green total factor productivity: Research on the spillover effect from China’s first-tier cities. Technol. Anal. Strateg. Manag..

[67] Xie J., Sun Q., Wang S., Li X., Fan F. (2020). Does Environmental Regulation Affect Export Quality? Theory and Evidence from China. Int. J. Environ. Res. Public Health.

[68] List J.A., Co C.Y. (2000). The Effects of Environmental Regulations on Foreign Direct Investment. J. Environ. Econ. Manag..

[69] Fan F., Lian H., Wang S. (2020). Can regional collaborative innovation improve innovation efficiency? An empirical study of Chinese cities. Growth Chang..

[70] Yu H.C., Liu Y., Liu C.L., Fan F. (2018). Spatiotemporal Variation and Inequality in China’s Economic Resilience across Cities and Urban Agglomerations. Sustainability.

[71] Shao S., Li X., Cao J.H. (2019). Urbanization Promotion and Haze Pollution Governance in China. Econ. Res. J..

[72] Elhorst J.P. (2014). Software for spatial panels. Int. Reg. Sci. Rev..

[73] LeSage J., Pace R.K. (2009). Introduction to Spatial Econometrics.

[74] Baron R.M., Kenny D.A. (1986). The Moderator-mediator Variable Distinction in Social Psychological Research: Conceptual, Strategic, and Statistical Considerations. J. Personal. Soc. Psychol..

